# Fine‐Grain Data Reveal Vulnerability of Biodiversity to Climate Change

**DOI:** 10.1111/gcb.70627

**Published:** 2025-12-09

**Authors:** Muyang Lu, Walter Jetz

**Affiliations:** ^1^ Ecology and Evolutionary Biology Yale University New Haven Connecticut USA; ^2^ Center for Biodiversity and Global Change Yale University New Haven Connecticut USA; ^3^ Key Laboratory of Bio‐Resources and Eco‐Environment of Ministry of Education, College of Life Sciences Sichuan University Chengdu Sichuan China; ^4^ Sichuan Key Laboratory of Conservation Biology on Endangered Wildlife, College of Life Sciences Sichuan University Chengdu Sichuan China

**Keywords:** climate change, hypervolume, niche, spatial scaling, species distribution models

## Abstract

Quantifying the impacts of ongoing climate change on biodiversity is one of the most pressing scientific challenges. Recent studies have indicated the risk of widespread range contraction and community collapse globally, but their specific interpretation and decision relevance are constrained by the coarse‐grain nature of their underlying evidence. Here, using a novel climate change vulnerability metric, we demonstrate for 1804 Western Hemisphere bird species that coarse‐grain estimates of climate change vulnerability show limited correspondence with those derived from biologically more relevant fine‐grain data. Coarse‐grain data used widely in recent assessments miss up to half of the most vulnerable species due to various degrees of underestimation and overestimation that covary with spatial autocorrelation and ecological attributes of species. As a result, the perceived vulnerability of high‐biodiversity tropical regions is heavily misrepresented, while temperate regions' vulnerability profiles remain relatively unaffected by this data limitation. For example, species in the Amazon region are even more vulnerable to climate change than previously reported. These findings alter the insights of recent global work and highlight the importance of considering the grain of available evidence. Informed collection of fine‐grain data combined with model‐based data fusion will be key for effectively assessing and managing the effects of climate change on biodiversity.

## Graphical abstract

This study finds that coarse‐grain data often misjudge species' climate change vulnerability, missing half of the most at‐risk species and misrepresenting tropical regions like the Amazon as less vulnerable. Fine‐grain data, paired with model‐based data fusion, is key to accurate biodiversity risk assessment and conservation.

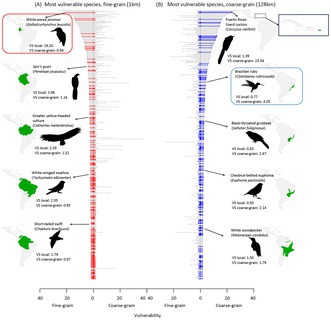

## 1 Introduction

Ongoing and future climate change poses an immense threat to biodiversity (Barnosky et al. 2011; Trisos et al. 2020; Urban 2015, 2024), and assessing how and where climatic change is affecting individual species is crucial to guiding effective conservation and management (Díaz et al. 2015; Hoffmann and Beierkuhnlein 2020; Trew and Maclean 2021). Ample evidence shows that species' environmental associations are undergoing accelerating perturbation and erosion—indicating widespread population declines and range contraction (Antão et al. 2022; Viana and Chase 2022). Robust avenues for identifying species and regions particularly vulnerable to climate change are thus key. For select species and specific locations, eco‐physiological and activity data have been able to support mechanistically informed, fine‐tuned projections (Kearney and Porter 2004; Riddell et al. 2021). But more comprehensive assessments tend to rely on large‐extent, coarse‐grain species distribution evidence to evaluate species' climatic niches and future risks (Murali et al. 2023; Pigot et al. 2023; Pinsky et al. 2019; Trisos et al. 2020). The widespread exposure to extreme climates (Murali et al. 2023; Pigot et al. 2023) and sudden collapses of global biodiversity (Pigot et al. 2023; Trisos et al. 2020) predicted by these studies have garnered notable attention. Their specific insights include that species in regions with limited climatic heterogeneity such as the Amazon basin face greater risks than those in mountains (Trisos et al. 2021). A key unaddressed aspect, however, is how much the coarse‐grain analysis accurately reflects a species' association with its living habitat and environment (Colwell 2021; Hurlbert and Jetz 2007; Jetz et al. 2019).

The dependence of environmental niche characterizations on spatial grain has long been recognized (Guisan et al. 2007; Nadeau et al. 2017; Wiens 1989), and there is growing appreciation that the strength and form of this link in turn vary with ecological factors (Connor et al. 2018; Lu and Jetz 2023; Mertes and Jetz 2018). In most work to date, the resolution of readily available environmental data has driven the spatial grain of analysis rather than the true spatial accuracy of biodiversity data or biological understanding (Lu and Jetz 2023). Critically, however, in the context of climate change, a mismatch between the grain size of relevant biological processes and that of evidence has the potential to cause biased vulnerability estimates and erroneous conservation insights (Cohen and Jetz 2025; Colwell 2021; Guisan et al. 2007). Exposure and vulnerability studies conducted at coarse spatial grain might only insufficiently and inconsistently capture the finer‐grain mechanisms of vulnerability and deliver results that are disconnected from the processes experienced by the organisms (Colwell 2021; Maclean and Early 2023; Scheffers et al. 2014). Although additional elevation or habitat information can be used to estimate habitat‐suitable ranges and decrease the false presence rate of expert range maps (Brooks et al. 2019; Powers and Jetz 2019; Smith et al. 2025), the effectiveness of this varies by species and resulting improvements to niche characterizations remain unclear.

Several empirical studies show that coarse‐grain data tend to overestimate species' sensitivity to climate change (Maclean and Early 2023; Meineri and Hylander 2017; Nadeau et al. 2022). The key insight underlying these findings is that, due to the spatial averaging effect of aggregating micro‐climatic data into coarse grains, the variance of environmental conditions across a species' range (and thus the estimated climatic tolerance of the species) decreases as grain size coarsens (Maclean and Early 2023). However, this argument relies on the assumption that species can occupy the full range of environmental conditions provided by microhabitats—effectively appearing as generalist species. Yet theory suggests the opposite pattern is equally likely (Lu and Jetz 2023). For example, coarse‐grain data could underestimate species' sensitivity to climate change if a species favors highly specialized microhabitats within a landscape (Lu and Jetz 2023). Thus, the direction of bias introduced by coarse‐grain analysis can go either way. To date, the lack of a comprehensive empirical evaluation has put into question both the magnitude of biodiversity risk from climate change and its specific geographic variation as asserted by recent work (Colwell 2021).

Here, we address this issue by assessing the grain‐size dependence of climate change vulnerability estimates for a comprehensive system, birds of the Western Hemisphere. The region's avifauna includes many already threatened species with large purported differences in climate change vulnerability and corresponding conservation recommendations (Bateman et al. 2020).

To further understand which niche axis drives the grain‐size dependence of climate change vulnerability estimates, we develop a novel partitioning framework using the hypervolumes of species' climatic niches (Hutchinson 1957). The approach characterizes species' climatic niches as following a multivariate normal distribution (Lu et al. 2021) and quantifies species' vulnerability to climate change by both its exposure (magnitude of climate change) and sensitivity (the niche breadth along the direction of climate change). Previous vulnerability measures have two key limitations: they either do not allow additive partitioning of the effects of different niche axes (Kling et al. 2020; Rinnan and Lawler 2019), or they unrealistically assume populations at the margin of a species' niche have the same sensitivity as populations at the niche center (Dickinson et al. 2015). Our proposed metric resolves the latter limitation by assuming that populations at the margin of a species' climatic niche space inherently have higher vulnerability than those at the niche center—because these marginal populations are already in suboptimal environmental conditions (Abeli et al. 2014).

We aim to answer two key questions in this study: (1) What are the magnitude and direction of bias in estimated climate change vulnerability introduced by coarse‐grain analysis? (2)

What are the biological and geographic drivers of the scale‐dependence of estimated climate change vulnerability?

## 2 Results

### 2.1 Coarse‐Grain Evidence Insufficiently Captures Fine‐Grain Vulnerability

**FIGURE 1:**
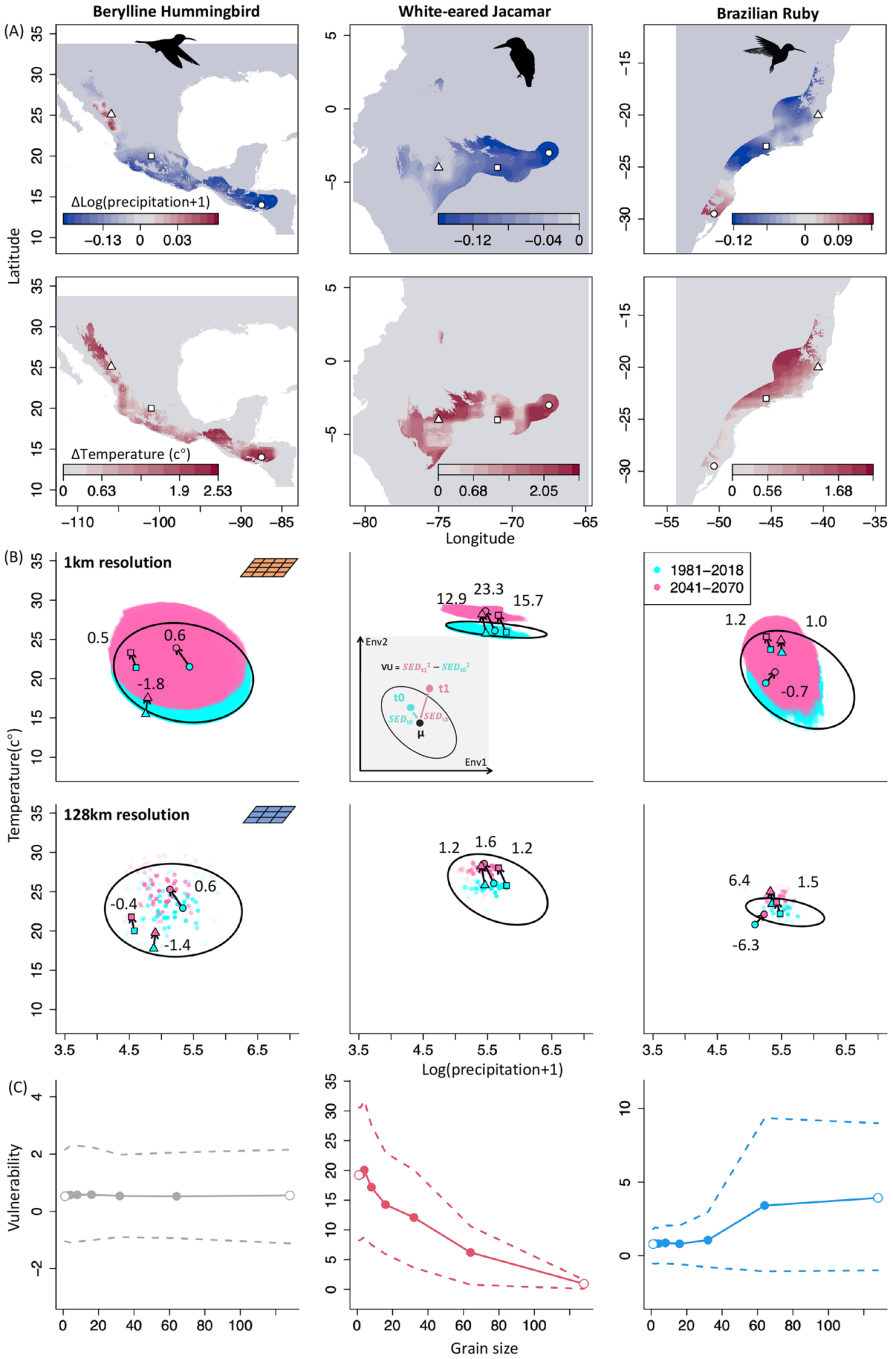
Grain size‐dependent climate change vulnerability in three New World bird species. (A) Projected change in temperature and precipitation between recent (1981–2018) and future (2041–2070) time periods, shown for fine‐grain (1 km; three example locations highlighted). Only pixels within the 80% quantile of the species' environmental niche are shown. The black contours show the outlines of the coarse‐grain (128 km) pixels covered by a species' range map buffered by 100 km. (B) Difference between recent (cyan points) and future (pink points) conditions shown in bivariate environment space and estimated from temporally annotated fine‐grain versus coarse‐grain occurrence data (1 km vs. 128 km grain). Black ellipses represent the 80% quantile of the species' environmental niches. These values serve as basis for calculating species‐level vulnerability, as showcased for the three example locations highlighted in (A). The vulnerability score (VS) of each grain is calculated as the difference between the squared standardized Euclidean distances (SED) between recent and projected environmental conditions to the niche center, as demonstrated by the inset figure in the middle panel of (B). For details on the calculation of the vulnerability score, see Figure 3. The vulnerability score (VS) also has a probabilistic interpretation: For a dimension count of two, an increase of VS from 0 to 0.45 is equivalent to a 20% drop of suitability relative to the suitability estimated at a species' niche center; an increase of VS from 0 to 1 is equivalent to a 40% drop of suitability and an increase of VS from 0 to 2 is equivalent to a 63% drop of suitability. (C) Mean vulnerability within a species' buffered range weighted by grain‐wise suitability plotted against grain size. Dashed lines show the 95% confidence intervals of the species‐level vulnerability.

**FIGURE 2:**
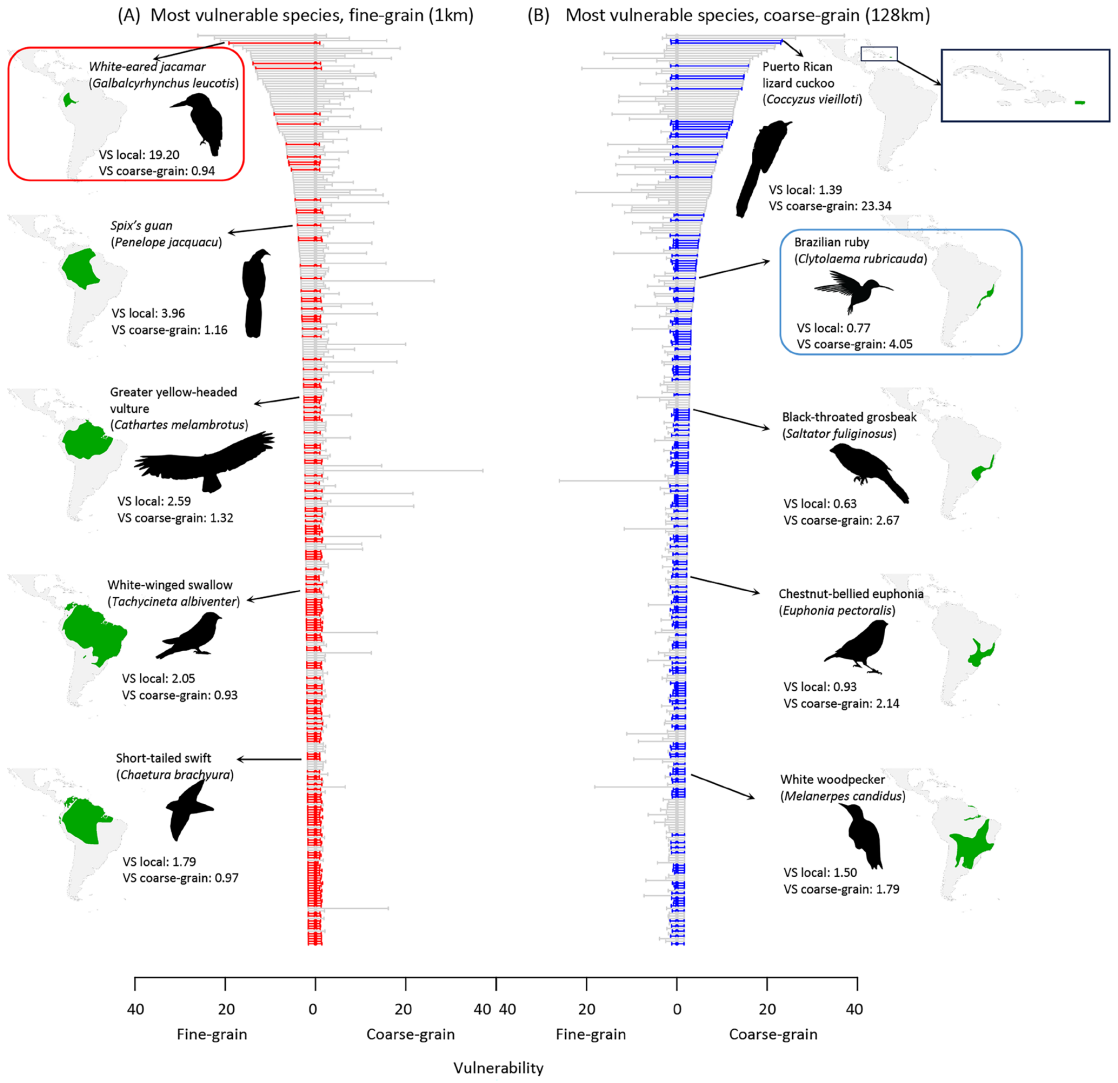
Discrepancy in species climate change vulnerability when assessed with fine‐grain (A) versus coarse‐grain (B) data. “VS” is short for “Vulnerability score.” The graphs list the top 20% of species for each grain size, ranked by vulnerability. Red and blue colors identify species that are among the top 20% most vulnerable species for only one but not the other grain size, and grey the species that are shared between two grain sizes. Five example species per grain size are highlighted with their range maps (green) shown for spatial context. Colored frames show the two example species in Figure 1: The White‐eared jacamar ( *Galbalcyrhynchus leucotis* ) and the Brazilian ruby ( *Clytolaema rubricauda* ).

**FIGURE 3:**
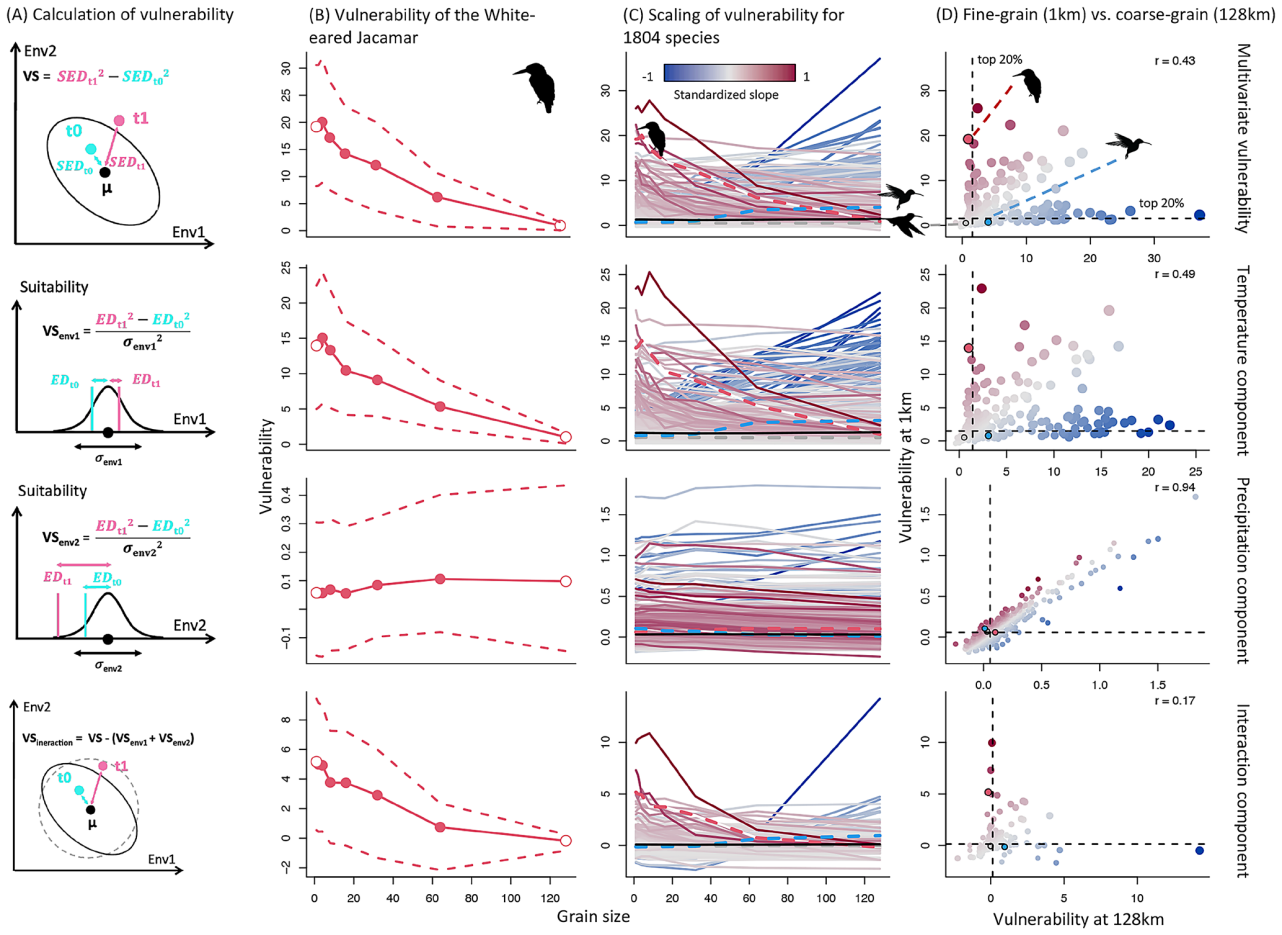
The scaling of vulnerability and its partitioned components. (A) The partitioning of the vulnerability of a site into univariate components and an interaction component. The inset in Figure 2 is shown in the first panel. *μ* represents the niche center, *t*_0_ represents the recent environmental condition and *t*_1_ represents the future environmental condition. The solid line ellipsoid represents the 95% quantile of the multivariate normal distribution of the climatic niche. The dashed circle represents the 95% quantile of a multivariate normal distribution where there is no interaction between niche axes. The vulnerability score is calculated as the difference between the squared standardized Euclidean distances (SED) of the current and projected future environmental conditions to the niche center. In the one‐dimensional case, it is the difference between the squared Euclidean distances (ED) divided by the one‐dimensional niche breadth (the variance, *σ* ^2^). When there is no interaction among niche axes (dashed circle), the multivariate vulnerability is just the sum of the univariate components. (B) Scaling of the multivariate vulnerability of the White‐eared Jacamar and its partitioned components across its geographic range (weighted by pixel suitability). Dashed lines show the 95% confidence intervals of vulnerability. (C) Vulnerability scaling expanded to all 1804 assessed species. Colored lines represent individual species, with red indicating negative and blue positive vulnerability‐scaling relationship and dashing marking the focal species shown in (B). Black solid lines show the average across all species. (D) Comparison of vulnerability based on fine‐grain versus coarse‐grain data 1804 species. Black dashed lines show the 80% quantiles of vulnerability at for each category, respectively.

Three example species illustrate the strongly different effects grain size has on projected climate change vulnerability (Figure 1). Conditions across the Berylline hummingbird (*Saucerottia beryllina*)'s range in the arid landscapes of Central America are projected to be significantly warmer and drier in ca. 30 years compared to today when characterized with fine‐grain data (1 km grain size). But relative to the species niche breadth at the same resolution (graphically represented as an ellipse in Figure 1B) this change appears moderate: the species vulnerability score is small (for calculation, see inset of Figures 1B and 3A), ca 0.5 (Figure 3C), implying a drop of ca. 20% in future suitability at the niche center. This vulnerability varies little with grain size and is similar for coarse‐grain data (50 km and coarser) of the sort used for large‐scale analyses (Trisos et al. 2020). This is different for the White‐eared jacamar ( *Galbalcyrhynchus leucotis* ) which, when analyzed with fine‐grain data, faces a climatic exposure that is substantial in relation to its narrow niche breadth. Its fine‐grain vulnerability score of 19 puts it into the top 0.2% of Western Hemisphere birds most threatened by climate change (Figure 2A). This understanding of climate risk, however, is progressively less appreciated with coarser grains: for coarse‐grain data its estimated vulnerability is up to 20 times lower putting the jacamar into the one‐third least affected species. This grain size dependence arises almost exclusively due to changes in niche breadth, with exposure itself showing relatively low grain size dependence (Figure 1B). The Brazilian ruby ( *Clytolaema rubricauda* ) shows the opposite pattern. Here, the coarse‐grain assessment suggests a large vulnerability of ca. 4, that is, among the top 25% of impacted species (Figure 2B). But analysis with ecologically more relevant fine‐grain data delivers a lower estimated vulnerability (Figure 1B). The species exhibits a positive vulnerability–grain size relationship. When analyzed with fine‐grain data, it has a vulnerability score of 0.77, placing it roughly in the bottom half of all species.

Across all 1804 assessed species, only about half of the most vulnerable species (the top 20%)—identified by either the 128 km coarse‐grain analysis or the 1 km fine‐grain analysis—are shared between the two sets (Figure 2). Overall, climate change vulnerability shows a highly heterogeneous scaling pattern (Figure 3C). Vulnerability assessments across the two grain sizes—separated by two orders of magnitude—are highly discrepant, with a Pearson correlation of only 0.43 between estimates from coarse‐grain and fine‐grain data (Figure 3D, Figure S1). Thirty percent of the 1804 species show a vulnerability difference of more than 0.45 between coarse‐grain and fine‐grain values (for context: a 0.45 increase in vulnerability score translates to a 20% reduction in suitability, relative to suitability at the niche center; see details in Section 4); for ca. 15% of the species, this vulnerability difference is more than 1 (corresponding to a 40% drop), and for 8% of the species, the vulnerability change is more than 2 (corresponding to a 70% drop). Partitioned into the core components, we find that temperature is the key driver of climate change vulnerability for our three example species (Figure 3B, Figures S2 and S3). We also find that temperature vulnerability contributes most strongly to overall vulnerability and to the observed grain size dependence for all assessed species (Figure 3C,D).

### 2.2 Biological and Landscape Factors Drive the Spatial Scaling of Vulnerability

**FIGURE 4:**
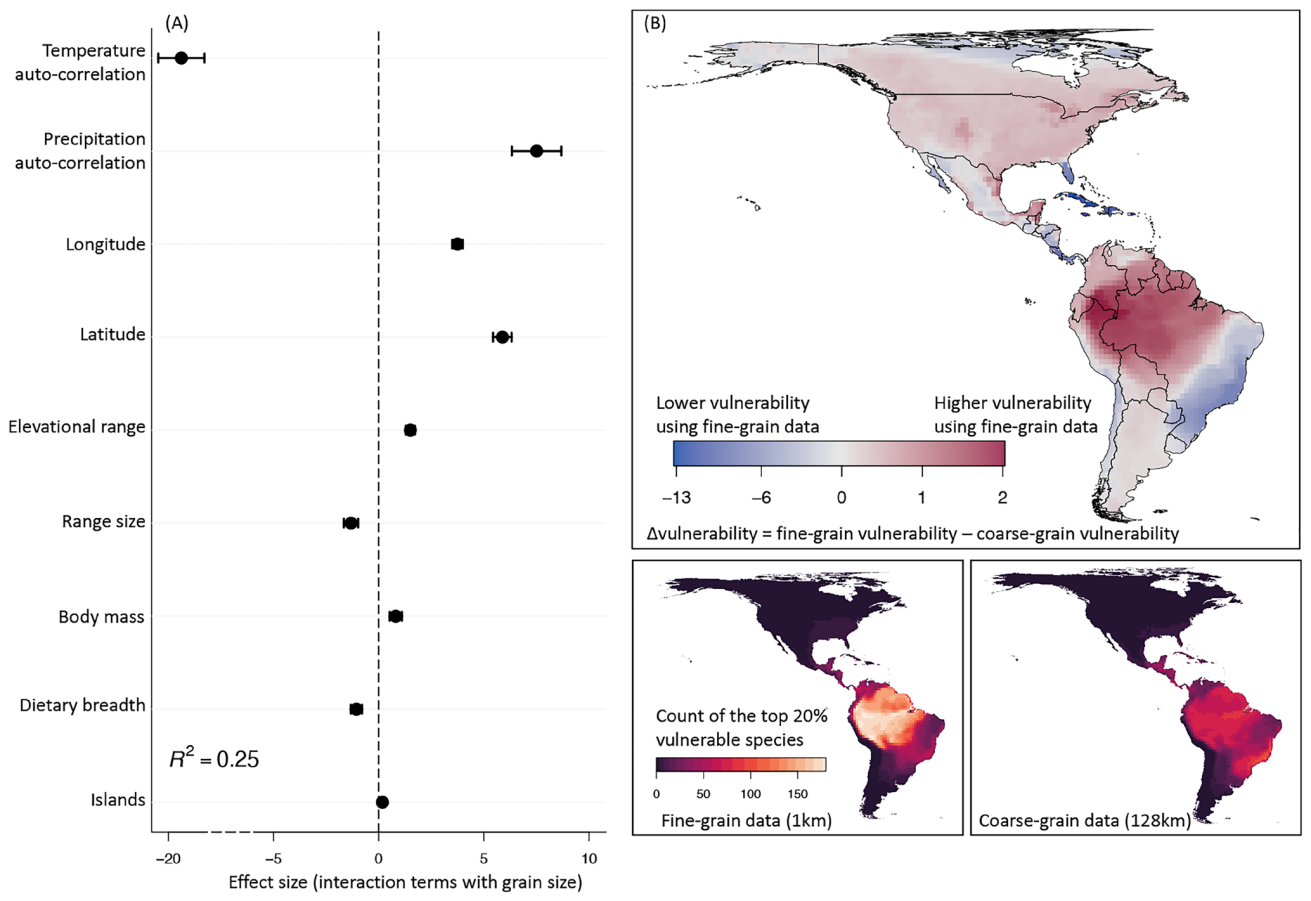
(A) The relative effects of nine hypothesized drivers on the spatial scaling of climate change vulnerability. A positive value means the larger the predictor is, the more likely the vulnerability is going to increase with grain size. A negative value means the larger the predictor is, the less likely the vulnerability is going to decrease with grain size. All nine predictors are combined in a single model. (B) Average difference (weighted by the inverse of range size) in species‐level vulnerability of 128 km assemblages when assessed with fine‐grain versus coarse‐grain data (fine‐grain vulnerability minus coarse‐grain vulnerability) based on1804 bird species (top panel). Negative values mean that coarse‐grain data overestimate, positive values mean that coarse‐grain data underestimate fine‐grain vulnerability. Bottom panels show the count of the top 20% vulnerable species based on fine‐grain (1 km) versus coarse‐grain data (128 km). Map lines delineate study areas and do not necessarily depict accepted national boundaries.

We find that a range of biological and landscape factors explains these scaling differences among species (Figure 4A). Temperature and precipitation auto‐correlation across the landscapes a species occurs in has by far the greatest effect (Figure 4A). In landscapes where nearby places have similar temperatures (e.g., places with low terrain variation), it is very likely that an analysis with coarse‐grain data misses species with high vulnerability. The opposite is true for precipitation: species in areas of strong geographic precipitation variability tend to be less vulnerable than coarse‐grain data might suggest. Other geographic and biological species attributes play a weaker role (Figure 4A). These results are robust across climate change models, scenarios, and spatial thinning distances of occurrence points (Figures S4–S9).

### 2.3 Tropical Regions Are More Vulnerable Than Temperature Regions When Assessed With Fine‐Grain Data

The strong role of landscape factors suggests that the grain size dependence of vulnerability varies geographically. Mapping the mean fine‐grain versus coarse‐grain vulnerability of assessed species, we find the tropics to be much more sensitive to grain size than temperate areas (Figure 4B). Specifically, the climate change vulnerability of species in the Amazon basin, the Atlantic Forest, and the Caribbean Islands is more likely to be impacted by the choice of grain size than species in other regions. There, up to 40% of species have an absolute change in vulnerability greater than 0.45 between fine and coarse grains (Figure S10). Accordingly, the hotspots of most vulnerable species differ substantially across grains (Figure 4B), with species in the Atlantic Forest emerging as less and those in the Amazon basin as more vulnerable when assessed with fine‐grain compared to coarse‐grain data.

## 3 Discussion

Despite the central importance of spatial scale in ecology and conservation (Levin 1992; Wiens 1989) and a growing recognition of the issue (Lembrechts et al. 2019; Zellweger et al. 2019), how spatial grain affects the assessment of species climate change vulnerability has yet to reach a consensus (Colwell 2021; Franklin et al. 2013; Maclean and Early 2023). We find that a significant portion of species show great grain size dependence in their vulnerability. In contrast to the conclusions drawn by some previous studies (Maclean and Early 2023; Nadeau et al. 2022), our study shows that the bias introduced by coarse‐grain analysis can go either way (Cohen and Jetz 2025) depending on species' attributes. These highly grain size‐sensitive species drive a very low congruence between the most vulnerable species identified with coarse‐grain versus fine‐grain data. At minimum, this suggests that estimates of species' climate change exposure and vulnerability are far less reliable than single‐grain‐size analyses alone would imply. But given that for almost all the assessed species, home range sizes and mechanistic response grains (Mertes and Jetz 2018) are not expected to exceed 1 km, this observed mismatch has additional, more concrete implications (Colwell 2021; Lembrechts et al. 2019; Lenoir et al. 2013). Research using coarse‐grain data may have missed important species and places with high exposure and vulnerability, while falsely drawing attention to species and areas that are in fact not as strongly affected. Specifically, we find that assemblages at the eastern slope of the Andes and in the Amazon basin might be even more vulnerable to climate change than recent studies suggested (Trisos et al. 2020, 2021), and some more coastal locations (e.g., the Atlantic Forests) potentially less impacted.

The finding that landscape factors such as environmental autocorrelation have a dominant effect highlights the major role of geographic context in robustly estimating climate change vulnerability. Depending on the nature of the variable considered, species in both heterogeneous and homogeneous landscapes can have highly grain size‐dependent climate change vulnerabilities, and so do species that are narrow‐ranged and habitat specialists. Such species are more likely to be found in the tropics than in temperate zones. The Amazon basin emerging as one of the most grain size‐sensitive regions might appear counter‐intuitive given its seemingly homogenous temperature and precipitation regime (Trisos et al. 2021). This is because high temperature autocorrelation could inflate coarse‐grain niche breadth estimates (Figure 3B) by reducing the occurrence density in suitable environmental conditions. It indicates that the forecasted abrupt impacts of climate change in this region (Trisos et al. 2020, 2021) might yet be underestimated (Colwell 2021).

When only temperature and precipitation are considered, temperature vulnerability emerges as the primary driver of multivariate vulnerability—outweighing both precipitation vulnerability and the interaction component. This result supports the widely accepted notion that warming poses particularly concerning threats to animals (Khaliq et al. 2014; Trisos et al. 2020). However, our findings do not discount the relevance of precipitation change, because precipitation still affects species indirectly, for example, through changes in vegetation structure and food sources (Klein et al. 2015).

Like most large‐scale studies, ours has several inherent limitations. However, some of these limitations likely lead us to underestimate the true extent to which grain size influences vulnerability estimates. For example, our analysis is restricted to species with sufficient occurrence data, but under‐sampled species are more likely to be rare, specialized, located in biodiversity hotspots (Meyer et al. 2016; Oliver et al. 2021) and therefore likely grain size‐sensitive. This suggests that our results might yet underestimate the importance of grain size. Further, as data availability limits us to 1 km as the finest grain, extending the assessment to yet finer grains and their microclimates will likely flag even greater cross‐grain inconsistencies and modulate some of the specific results (Maclean and Early 2023; Zellweger et al. 2020). An underestimation of grain size sensitivity would also arise if important climatic variables were missed in our analysis, because higher niche dimensions increase the chance for including grain size‐sensitive niche axes to enter the vulnerability assessment. The assumption of a multivariate normal distribution of species niche variables might similarly cause an underestimation of species' true sensitivity to climate change, because many species have a warm‐skewed temperature response curve (Pigot et al. 2023) or high intra‐specific niche variation (Carlson et al. 2021) which could lead to stronger grain size sensitivity. The consideration of these and other factors such as habitat specialization, dispersal ability, and evolutionary adaptation, and lagged climatic response are all expected to modulate niche‐based climate change assessment, but would not alter the general finding of the grain‐size dependence of vulnerability estimates. Lastly, our analysis is limited by the inherent accuracy of the environmental data products themselves. Different data products can yield divergent predictions of environmental variables, and these discrepancies vary geographically—likely being more pronounced in regions with sparse weather stations (Karger et al. 2017). Given that high‐elevation regions generally have lower weather station density than low‐elevation regions, the environmental heterogeneity and true extent of scale sensitivity in species' vulnerability in mountain systems may be underestimated by our current environmental products. Notably, temperature is generally more accurately represented in downscaled environmental products than precipitation (Behnke et al. 2016)—a bias that may explain the low contribution of the precipitation niche component to the grain dependence of climate change vulnerability observed in our results. Additionally, the accuracy of climate change projections (e.g., CMIP6) further impacts our quantification of the exposure component in vulnerability assessments (E. Williams et al. 2024). Such discrepancy might result in less accurate vulnerability assessment of species in the tropical regions. Broadly, the results presented here firmly illustrate how the recent generation of near‐global coarse‐grain findings will need to be updated with work that addresses the uncovered biases from grain size‐dependent vulnerabilities. We suggest that doing so will require a combination of grain‐conscious additional sampling, model‐supported fusion of different data types, mechanism‐informed identifications of optimal analysis grains (Jackson and Fahrig 2015), or multi‐grain analytical approaches (Mertes and Jetz 2018).

Evidently less limited by gaps and biases of incidental point records (Jetz et al. 2019; Oliver et al. 2021), expert range maps offer a strong avenue to address the geographic distribution of speciose taxa globally (Jetz, McPherson, and Guralnick 2012). But their typically high and ecologically non‐random false presence rates when analyzed at resolutions < 100–150 km mean the grain size‐dependence issue we demonstrated here is necessarily incurred (Hurlbert and Jetz 2007). Even as some recent global studies might have chosen 25–50 km grains for analysis, this does not alter the coarse‐grain nature of the signal underpinning expert map data (Hurlbert and Jetz 2007). In this case, inferring environmental niches at a finer grain than the expert maps allows for more false presences which could seriously underestimate the climate change sensitivity of specialized species in highly heterogeneous landscapes. We find that smaller grain size differences (e.g., 30–50 times smaller), can still have vast effects on estimated vulnerabilities (Figure S1), implying that even when captured accurately, occupancy at 25–50 km grain might not recover true fine grain sensitivity. Identifying the optimal grain for each species based on their behavioral and ecological attributes is challenging (Mertes and Jetz 2018). Here we show that analyzing species grain size‐sensitivity alone provides valuable means to address the issue of grain‐dependence: for grain size‐insensitive species, vulnerability assessment could be reliably conducted regardless of the underlying grain size; while for grain size‐sensitive species, at minimum identifying the range of grain sizes that yield robust estimates and potential sensitivity analyses is key. For example, in the White‐eared jacamar, grain sizes smaller than 20 km keep the difference in vulnerability estimates to at most 25% compared to 1 km measurements, while using a 50 km grain size would change the vulnerability estimate by 50%.

Our results highlight the importance of additional collection of fine‐grain data (Gonzalez et al. 2023; Oliver et al. 2021), and could help in prioritizing the species, regions, and locations in greatest need of fine‐grain climate change‐relevant evidence. Alongside, fusion of different biodiversity data types and models can assist in overcoming their accuracy–coverage trade‐offs (Jetz et al. 2019; Meineri and Hylander 2017) and in gauging the potential effect of grain size dependence (Jetz et al. 2019). A combination of informed field data collection and robust quantitative tools will be key to effectively safeguarding biodiversity in a rapidly changing world.

## 4 Methods and Materials

### 4.1 Occurrence Data

We obtained species' occurrence data from eBird (Sullivan et al. 2009). We filtered eBird data to those that are observed within the breeding seasons (we use May to July as the approximate breeding season for the Northern Hemisphere; November to January for the Southern Hemisphere) between 1981 and 2018 (38 years) and within 1 km traveling distance from the recorded coordinates. To reduce potential spatial biases, we further thinned occurrences to be at least 5 km apart with R package “spThin” (Aiello‐Lammens et al. 2015). Results using 10 and 20 km thinning distances are in Supporting Information (Figures S5 and S6). We only kept species with more than 20 occurrence records after thinning for further analysis, resulting in a final dataset of 1804 species comprising 860,186 records representing the breeding seasons for these 38 years. We also obtained species' expert range map from Jetz, Thomas, et al. (2012) to determine modeling extent and calculate range‐map derived attributes (range size, centroid latitude and longitude, spatial autocorrelation). Note that for any species' climatic niche characterizations eBird based point records were used, not expert maps (see below Sections 4.4 and 4.5).

### 4.2 Environmental Data

We focused our niche assessment on temperature and precipitation, core climatic drivers of ecological processes (Jetz and Rahbek 2002; Kreft and Jetz 2007) that were also the focus of prominent work addressing the biodiversity consequence of climate change (Murali et al. 2023; Pigot et al. 2023; Trisos et al. 2020). We chose these two variables also because they are the only variables that have high temporal resolution that can be used to extract environmental information at the time when an occurrence was recorded. We used CHELSA V2.1 data at 30″ (~1 km) resolution (Karger et al. 2017, 2021) to derive a monthly timeseries for 1981 and 2018 and monthly averages for 2041 to 2070 based on two different CMIP6 models (GFDL‐ESM4 and IPSL‐CM6A‐LR) and are used for SSP126, SSP370 and SSP585 scenarios. The results for GFDL‐ESM4 SSP370 are shown in the main text, and the rest is provided in the Supporting Information (Figures S7–S9). We aggregated the environmental layers to 8 different resolutions (1, 2, 4, 8, 16, 32, 64 and 128 km) so that the coarsest resolution corresponds roughly to the accuracy of species' expert range maps (Hurlbert and Jetz 2007).

### 4.3 Species Attributes

To quantify range size, centroid latitude, centroid longitude, and spatial heterogeneity within its range, we used expert range maps from Jetz, Thomas, et al. (2012). We first buffered both the range maps and the occurrence points by 0.5° (~50 km). Then we merged the buffered range maps and the buffered occurrence points to produce an updated range map. This procedure is to ensure that the range maps used in the subsequent analysis include all the fine‐grain occurrence points. The procedure slightly inflates the range size, but the results are robust when we used the original range maps to calculate the range size (Figure S11). In our calculation of species vulnerability, we also use a weighting method to ensure that the inflation of range size does not affect species‐level estimates (see Section 4.4). We then used the buffered range map to calculate species geographic range size, absolute centroid latitude, and longitude. We also used the map to calculate spatial heterogeneity characteristics of the landscape occupied by species using Moran's *I* within a 10 km distance for both the average temperature and average precipitation during the breeding seasons between 1981 and 2010. Moran's *I* is a coefficient of spatial autocorrelation, which we used as an inverse measure of spatial heterogeneity. Higher spatial autocorrelation represents lower spatial heterogeneity.

For 1759 of the 1804 total species we obtained data on mean and range of elevations from an independent dataset compiled by Quintero and Jetz (2018). For the remaining 45 species whose information was not covered by Quintero and Jetz (2018), we derived this information from an overlay of their occurrence records with a global elevation layer (Amatulli et al. 2018). Dietary niche breadth and elevational range were used as proxies for habitat specialization. To account for the influence of species' occurrence on islands, we calculated the proportion of each species' occurrence points located on Caribbean islands and denoted this variable as “island.” We obtained species body mass and dietary data from the EltonTrait database (Wilman et al. 2014). Body mass was log transformed, and dietary breadth was calculated by Levin's niche breadth index (Feinsinger et al. 1981), *B*.

$$B=1/R\sum_{i}{p_{i}}^{2}$$

where *R* is the number of dietary categories, *p*_*i*_ is the relative frequency of the corresponding dietary category.

### 4.4 Vulnerability Measure

We developed a climate change vulnerability metric that incorporates sensitivity and exposure in the multivariate environmental space. Our metric allows additively partitioning a multivariate vulnerability score into its univariate components and an interaction component. A major advantage of this method is that beyond the consideration of the magnitude of exposure, as provided by alternative popular measures (Dickinson et al. 2015; Rinnan and Lawler 2019; J. W. Williams et al. 2007) it also addresses the position and direction of exposure in the environmental space (Kling et al. 2020; Mahony and Cannon 2018).

Consider a one‐dimensional environmental space, denote *x*_*i,t*0_ as the environmental condition of location *i* at the current time *t*_0_, and *x*_*i,t*1_ as the environmental condition of location *i* in the future time *t*_1_. A simple climate change vulnerability score (VS) for the species at location *i* is the Euclidean distance (ED) between future and current climate, standardized by the species' niche breadth *σ* (J. W. Williams et al. 2007):

(1) $${\mathrm{VS}}_{i,\text{naive}}=\sqrt{\frac{{\left(x_{i,t1}-x_{i,t0}\right)}^{2}}{\sigma^{2}}}$$

where the numerator |*x*_*i*,*t*1_ − *x*_*i*,*t*0_| measures the climate change exposure, and the denominator *σ* measures species' sensitivity to climate change. There are two major issues with this naïve metric: (1) by always assuming a positive number, it implies that climate change is always harmful to a species regardless of whether the species' local suitability increases or decreases; (2) it assumes that exposure always has the same effect no matter whether the local population is in a favorable environment or an unsuitable environment. A realistic vulnerability measure should (1) allow the local vulnerability to decrease if the condition becomes more favorable in the future; and (2) predict higher risk for populations that are already in unfavorable environments given the same exposure because they are already stressed (Abeli et al. 2014; Margalef‐Marrase et al. 2020; Sexton et al. 2009). To achieve these goals, we propose a new vulnerability measure modified from Equation (1):

(2) $${\mathrm{VS}}_{i}={\mathrm{SED}}_{t1}^{2}-{\mathrm{SED}}_{t0}^{2}=\frac{{\left(x_{i,t1}-\mu \right)}^{2}}{\sigma^{2}}-\frac{{\left(x_{i,t0}-\mu \right)}^{2}}{\sigma^{2}}$$

where *μ* is the species' niche center. This metric quantifies the difference between two squared standardized Euclidean distances (SED): the SED between the current climatic condition at location *i* and the species' niche center, and the SED between the future climatic condition at location *i* and the species' niche center. By assuming that a species' niche center is most suitable for a population (de la Fuente et al. 2021; Osorio‐Olvera et al. 2020), this metric predicts that vulnerability will increase if climate change is moving the local population away from the niche center, and decrease if it is moving the local population toward the niche center. This metric also predicts higher risk for populations at the margin of the climatic niche. For example, raising the temperature by 1 (assuming *σ* = 1 for demonstration), a population at the center of its niche will have a vulnerability score of 1^2^–0^2^ = 1, while a population that's 2 *σ*s away from the center will have a vulnerability score of 3^2^–2^2^ = 5.

When generalized to the n‐dimensional climatic space, the standardized Euclidean distances in Equation (2) are equivalent to the Mahalanobis distances (Mahony et al. 2017), which are essentially standardized Euclidean distances calculated with principal components:

(3) $$\begin{array}{c} {\mathrm{VS}}_{i}={\mathrm{SED}}_{t1}^{2}-{\mathrm{SED}}_{t0}^{2}={\left(x_{i,t1}-\mu \right)}^{T}\Sigma^{-1}\left(x_{i,t1}-\mu \right)\\ -{\left(x_{i,t0}-\mu \right)}^{T}\Sigma^{-1}\left(x_{i,t0}-\mu \right)\\ ={\mathrm{MD}}_{t1}^{2}-{\mathrm{MD}}_{t0}^{2}=∆MD^{2} \end{array}$$

where *x*_*i*,*t*1,_ *x*_*i*,*t*0_ and *μ* are vectors of environmental variables with length *n*, and Σ is an n by n covariance matrix that captures a species' multidimensional niche breadth. Using a novel partitioning framework, the Mahalanobis distances can be written as the sum of univariate SED and an interaction component. The interaction component measures how much the correlations among niche axes further decrease or increase the summed standardized Euclidean distance between an environmental condition and the niche center (Lu et al. 2021). It is named the “interaction” component because given a one‐dimensional exposure, the consideration of an additional niche dimension could exacerbate or alleviate the sum of uni‐dimensional vulnerability. Take a two‐dimensional climatic space for example, and let *x* and *y* be the two climatic variables; the squared Mahalanobis distance between the local climate and species' niche center is:

(4) $$\begin{array}{c} MD^{2}=\frac{{d_{x}}^{2}}{\sigma_{x}^{2}}+\frac{{d_{y}}^{2}}{\sigma_{y}^{2}}+\frac{1}{\left|\Sigma \right|}\left(\rho^{2}{\left(d_{x}\sigma_{x}-d_{y}\sigma_{y}\right)}^{2}+2\rho d_{x}d_{y}\sigma_{x}\sigma_{y}\left(\rho -1\right)\right)\\ ={\mathrm{MD}}_{x}^{2}+{\mathrm{MD}}_{y}^{2}+{\mathrm{MD}}_{\text{interaction}}^{2} \end{array}$$

where $d_{x}=x-\mu$, $d_{y}=y-\mu$, *ρ* is the correlation between *x* and *y* in the covariance matrix Σ, and $\left|\Sigma \right|$ is the determinant of the covariance matrix. Substituting Equation (4) into Equation (3), the climate change vulnerability for a two‐dimensional climatic space is:

(5) $$\begin{array}{c} VS_{i}={\mathrm{MD}}_{t1}^{2}-{\mathrm{MD}}_{t0}^{2}=∆MD^{2}\\ =VS_{x}+VS_{y}+VS_{\text{interaction}}\\ =\frac{{d_{x,t1}}^{2}}{\sigma_{x}^{2}}-\frac{{d_{x,t0}}^{2}}{\sigma_{x}^{2}}+\frac{{d_{y,t1}}^{2}}{\sigma_{y}^{2}}-\frac{{d_{y,t0}}^{2}}{\sigma_{y}^{2}}\\ +\frac{1}{\left|\Sigma \right|}\left(\rho^{2}{\left(d_{x,t1}\sigma_{x}-d_{y,t1}\sigma_{y}\right)}^{2}+2\rho d_{x,t1}d_{y,t1}\sigma_{x}\sigma_{y}\left(\rho -1\right)\right)\\ -\frac{1}{\left|\Sigma \right|}\left(\rho^{2}{\left(d_{x,t0}\sigma_{x}-d_{y,t0}\sigma_{y}\right)}^{2}+2\rho d_{x,t0}d_{y,t0}\sigma_{x}\sigma_{y}\left(\rho -1\right)\right) \end{array}$$

Which partitions multivariate vulnerability into univariate components and an interaction component, therefore allowing us to compare their relative importance (Figure 3A).

The species‐level climate change vulnerability was calculated by averaging the local vulnerability across the grains within a species' range (buffered by 0.5°). Note that such a calculation does not consider the effect of dispersal. A high vulnerability score entails more cost for the species to disperse or adapt. Therefore, it does not measure the actual extinction risk. Because of the high false presence rate within the range maps at finer grains (Hurlbert and Jetz 2007) (and because the updated range maps we used in the analysis also include more unsuitable area), the unsuitable locations will be overly represented by Equation (4). To account for this false presence, we weighed the vulnerability of each location *i* by its climatic suitability:

(6) $$E\left(\mathrm{VS}\right)=\sum_{i}^{k}\frac{w_{i}}{\sum w_{i}}{\mathrm{VS}}_{i}$$

In this paper, we used the suitability at time *t*_0_ derived from the kernel of a Gaussian distribution (Etherington 2019) as weights:

(7) $$w_{i}=e^{-{\left(x_{i,t0}-\mu \right)}^{T}\Sigma^{-1}\left(x_{i,t0}-\mu \right)}$$

This suitability ranges from 0 to 1, with 1 representing the suitability at the species' niche center (assumed to be the optimal condition).

This procedure effectively controls for the effect of false presence because sites with false presence probability are likely to occupy less suitable climatic conditions. They will be assigned low suitability scores and therefore lower weights in the species‐level vulnerability score. Biologically, because the normalized suitability score represents the relative occurrence rate of a species at a site (Merow et al. 2013), Equation (6) can be interpreted as the expected vulnerability across a species' geographic range.

Because the Mahalanobis distance follows a chi‐square distribution in which the degrees of freedom equal the total number of dimensions (Etherington 2019), our vulnerability score (VS) also has a probabilistic interpretation: when the dimension is two, an increase of VS from 0 to 0.45 corresponds to a 20% drop in suitability relative to the suitability estimated at a species' niche center. The value of 20% is obtained by the probability of observing a Mahalanobis distance with less than 0.45 when degrees of freedom equal 2. This calculation assumes that the niche center has the highest suitability, and suitability decreases as distance from the niche center increases. Similarly, an increase of VS from 0 to 1 corresponds to a 40% drop in suitability and an increase of VS from 0 to 2 corresponds to a 63% drop in suitability.

### 4.5 Statistical Analysis

For each species, we extracted for each occurrence record the mean temperature and precipitation during the breeding seasons of the recorded year (this accounts for annual temporal variation in climatic conditions). By assuming that species niche preference remained unchanged between 1981 and 2018 and species completely tracked their niches over time, a species' environmental niche center *μ*, and the covariance matrix Σ were estimated by the average of 100 random sets of occurrence points spatially thinned by at least 5 km apart to reduce spatial bias (with occurrences matched to the observed years). We then calculated the climate change vulnerability using the 30‐year average temperature and precipitation between 1981 and 2018 as *x*_*t*0_, and the projected average climate between 2041 and 2070 as *x*_*t*1_ (Equations (3), (4), (5)). Species‐level vulnerability was calculated as the average pixel‐level vulnerability within the buffered range weighted by pixel‐level suitability (Equations (6), (7)). We calculated the species‐level vulnerability for all 1804 species at eight grain sizes. We used correlation plots to demonstrate the pairwise comparison between species‐level vulnerability calculated at different grain sizes.

We used a linear mixed effect model to examine the grain size dependence of species‐level vulnerability (Bates et al. 2015). The species‐level weighted mean vulnerability across a species' geographical range was the response variable. We modeled grain size, range size, temperature autocorrelation, precipitation autocorrelation, elevational range, mean elevation, body mass, dietary niche breadth, proportion of points on islands, centroid latitude, and longitude, and the interaction terms between grain size and all previously mentioned predictors as fixed effects. Species identity was included as a random effect on the intercept.

To show the spatial pattern of the grain size dependence of species‐level climate change vulnerability, we mapped the mean grain size dependence for species distributed within each 128 km grid cell, weighted by the inverse of range size to control for the effect of wide‐ranged species. The grain size dependence for each species was calculated as the difference between vulnerability calculated at the 1 km grain and 128 km grain. A positive value means that the fine grain vulnerability estimate is larger than the coarse grain vulnerability estimation; a negative value means the opposite. We also mapped the number of the most vulnerable species (defined as the 20% most vulnerable species) quantified at 1 km grain and 128 km grain.

Behrmann equal‐area projection was used for mapping.

## Author Contributions

**Muyang Lu:** conceptualization, data curation, formal analysis, investigation, methodology, writing – original draft, writing – review and editing. **Walter Jetz:** conceptualization, funding acquisition, project administration, resources, supervision, writing – original draft, writing – review and editing.

## Funding

This work was primarily supported by the Max Planck‐Yale Center for Biodiversity and Global Change and by the E.O. Wilson Biodiversity Foundation in furtherance of the Half‐Earth Project. M.L. is also supported by “the Fundamental Research Funds for the Central Universities” at Sichuan University, which facilitated the completion of this work.

## Conflicts of Interest

The authors declare no conflicts of interest.

## Supporting information


**Appendix S1:** gcb70627‐sup‐0001‐AppendixS1.docx.

## Data Availability

All datasets used here are publicly available. The occurrence data are available from the eBird website https://science.ebird.org/en/use‐ebird‐data. The climatic data are available from the CHELSA website https://chelsa‐climate.org. Trait datasets are available from https://figshare.com/collections/EltonTraits_1_0_Species‐level_foraging_attributes_of_the_world_s_birds_and_mammals/3306933. Code availability: Computer codes to repeat the analysis are available on Code Ocean (https://doi.org/10.24433/CO.3365464.v1).
