# Supplementary material for: Fine‐Grain Data Reveal Vulnerability of Biodiversity to Climate Change
Source: Glob Chang Biol. 2025 Dec 9;31(12):e70627. doi: 10.1111/gcb.70627 (PMC12687110; doi:10.1111/gcb.70627)
Supplement: Supplementary file 1 — Appendix S1: gcb70627‐sup‐0001‐AppendixS1.docx. [file GCB-31-e70627-s001.docx]

**Supporting Information. Extended Data Figures**


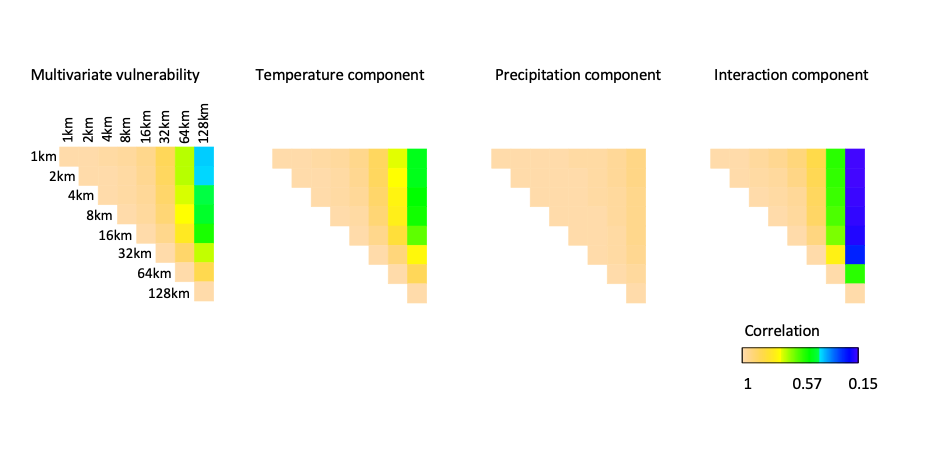


Extended Data Figure 1. Pairwise correlation between vulnerability score calculated at different grain size for the 1809 species. The multivariate vulnerability score equals the sum of the three partitioned components.


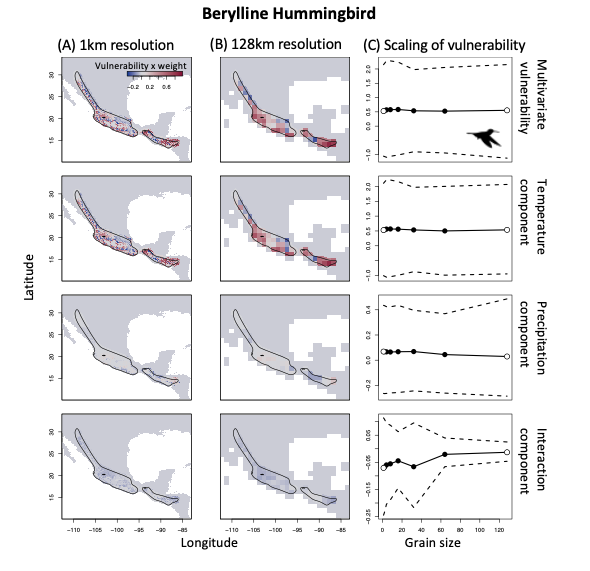


Extended Data Figure 2. (A) Maps of vulnerability multiplied by suitability at the 1km resolution for the Berylline hummingbird (*Saucerottia beryllina*). (B) Maps of vulnerability multiplied by suitability at the 128km resolution. (C) Mean multivariate vulnerability as well as the partitioned components within a species’ range weighted by suitability plotted against grain size. Dashed lines show the 95% confidence intervals of weighted vulnerability.


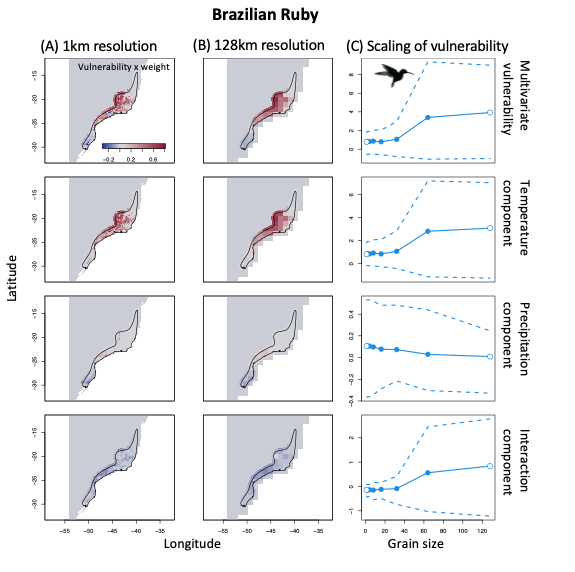


Extended Data Figure 3. (A) Maps of vulnerability multiplied by suitability at the 1km resolution for the Brazilian Ruby (*Clytolaema* *rubricauda*). (B) Maps of vulnerability multiplied by suitability at the 128km resolution. (C) Mean multivariate vulnerability as well as the partitioned components within a species’ range weighted by suitability plotted against grain size. Dashed lines show the 95% confidence intervals of weighted vulnerability.


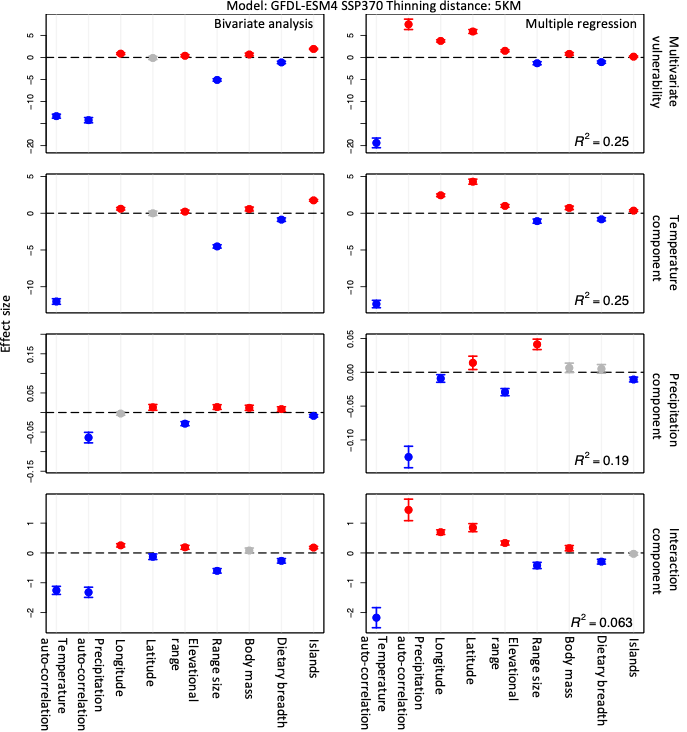


Extended Data Figure 4. Effect size of predictors on the slope of vulnerability-grain size relationships (interaction terms between predictor and grain size) using linear mixed effect models. The projection model is GFDL-ESM4 SSP370. 5km spatial thinning is used. There are in total 1804 species in the analysis. The first row shows models using the multivariate vulnerability as the response variable. The second to the fourth row shows models using the partitioned components as response variables. Species’ ID is modeled as a random effect on the intercept. x-axis shows the predicting variables. The left column shows the results of the bivariate analysis (vulnerability as response, grain size, one covariate, and their interaction term as predictors). The right column shows the results of the multivariate analysis.


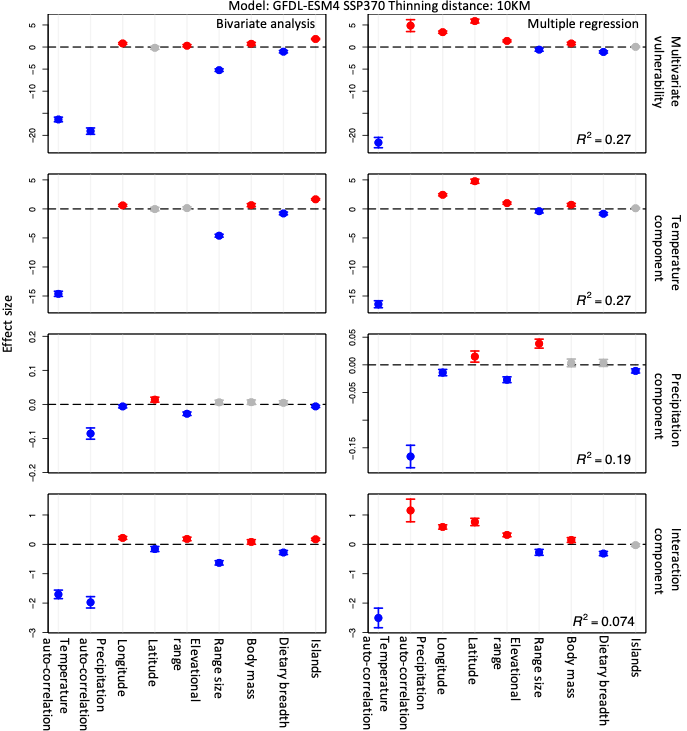


Extended Data Figure 5. Effect size of predictors on the slope of vulnerability-grain size relationships (interaction terms between predictor and grain size) using linear mixed effect models. The projection model is GFDL-ESM4 SSP370. 10km spatial thinning is used. There are in total 1703 species in the analysis. The first row shows models using the multivariate vulnerability as the response variable. The second to the fourth row shows models using the partitioned components as response variables. Species’ ID is modeled as a random effect on the intercept. x-axis shows the predicting variables. The left column shows the results of the bivariate analysis (vulnerability as response, grain size, one covariate, and their interaction term as predictors). The right column shows the results of the multivariate analysis.


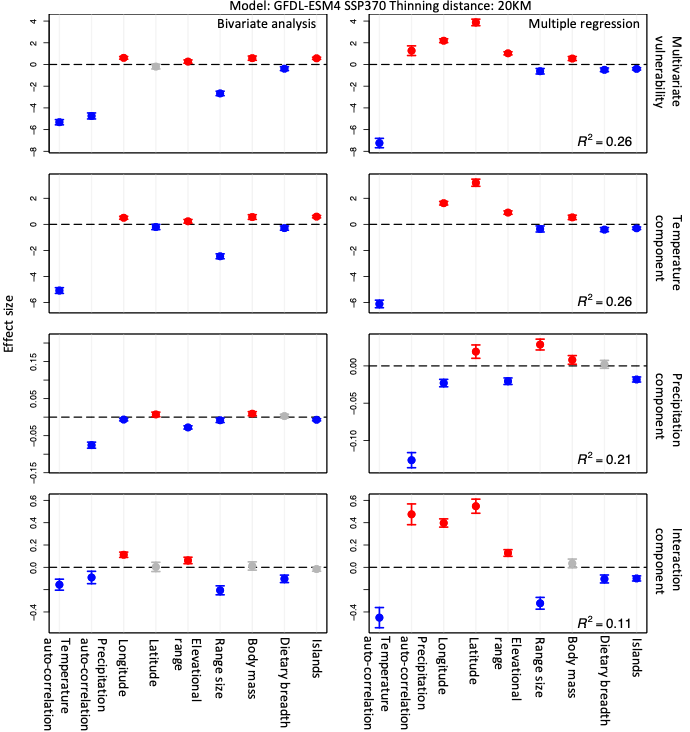


Extended Data Figure 6. Effect size of predictors on the slope of vulnerability-grain size relationships (interaction terms between predictor and grain size) using linear mixed effect models. The projection model is GFDL-ESM4 SSP370. 20km spatial thinning is used. There are in total 1546 species in the analysis. The first row shows models using the multivariate vulnerability as the response variable. The second to the fourth row shows models using the partitioned components as response variables. Species’ ID is modeled as a random effect on the intercept. x-axis shows the predicting variables. The left column shows the results of the bivariate analysis (vulnerability as response, grain size, one covariate, and their interaction term as predictors). The right column shows the results of the multivariate analysis.


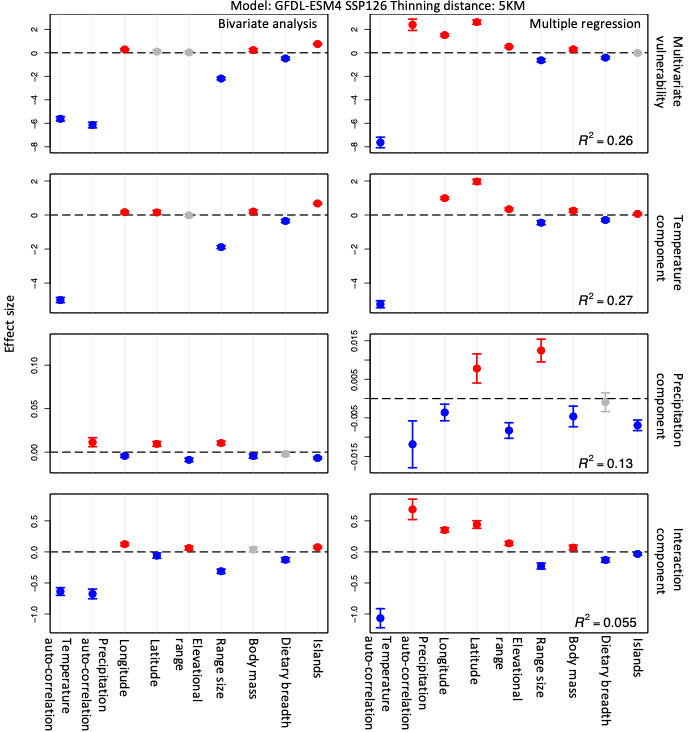


Extended Data Figure 7. Effect size of predictors on the slope of vulnerability-grain size relationships (interaction terms between predictor and grain size) using linear mixed effect models. The projection model is GFDL-ESM4 SSP126. 5km spatial thinning is used. There are in total 1804 species in the analysis. The first row shows models using the multivariate vulnerability as the response variable. The second to the fourth row shows models using the partitioned components as response variables. Species’ ID is modeled as a random effect on the intercept. x-axis shows the predicting variables. The left column shows the results of the bivariate analysis (vulnerability as response, grain size, one covariate, and their interaction term as predictors). The right column shows the results of the multivariate analysis.


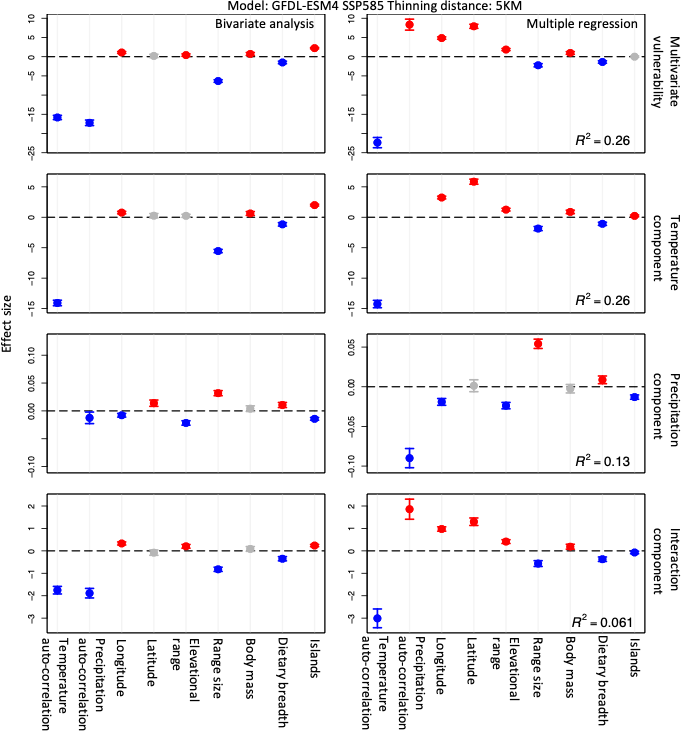


Extended Data Figure 8. Effect size of predictors on the slope of vulnerability-grain size relationships (interaction terms between predictor and grain size) using linear mixed effect models. The projection model is GFDL-ESM4 SSP585. 5km spatial thinning is used. There are in total 1804 species in the analysis. The first row shows models using the multivariate vulnerability as the response variable. The second to the fourth row shows models using the partitioned components as response variables. Species’ ID is modeled as a random effect on the intercept. x-axis shows the predicting variables. The left column shows the results of the bivariate analysis (vulnerability as response, grain size, one covariate, and their interaction term as predictors). The right column shows the results of the multivariate analysis.


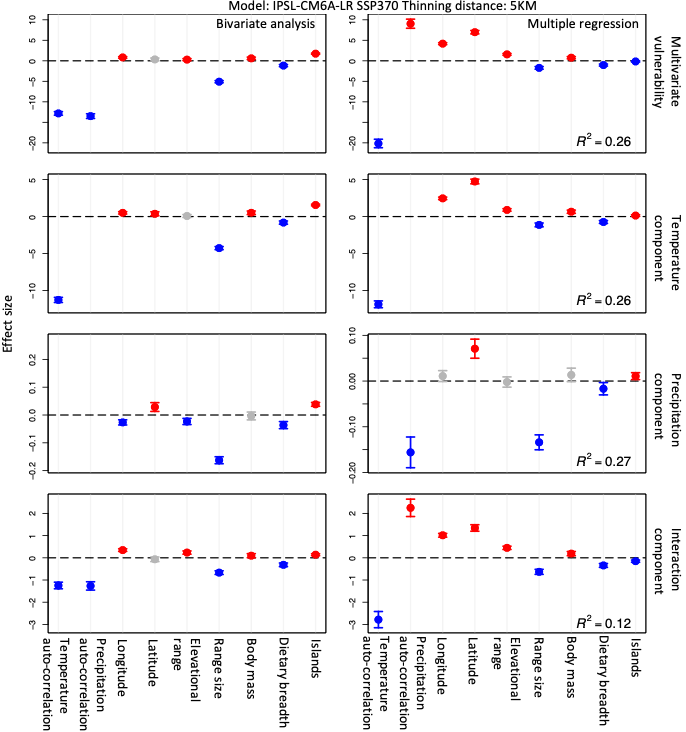


Extended Data Figure 9. Effect size of predictors on the slope of vulnerability-grain size relationships (interaction terms between predictor and grain size) using linear mixed effect models. The projection model is ISPL-CM6A-LR SSP370. 5km spatial thinning is used. There are in total 1804 species in the analysis. The first row shows models using the multivariate vulnerability as the response variable. The second to the fourth row shows models using the partitioned components as response variables. Species’ ID is modeled as a random effect on the intercept. x-axis shows the predicting variables. The left column shows the results of the bivariate analysis (vulnerability as response, grain size, one covariate, and their interaction term as predictors). The right column shows the results of the multivariate analysis.


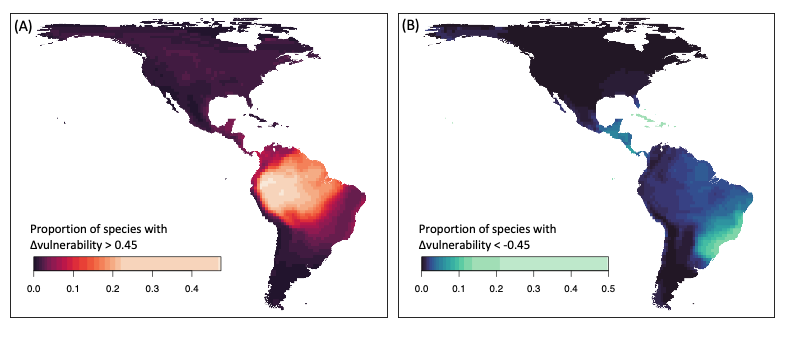


Extended Data Figure 10. (A) Proportion of species with ∆vulnerability (fine-grain vulnerability minus coarse-grain vulnerability) > 0.45 in the 128 km grid cells. (B) Proportion of species with ∆vulnerability < -0.45 in the 128 km grid cells.


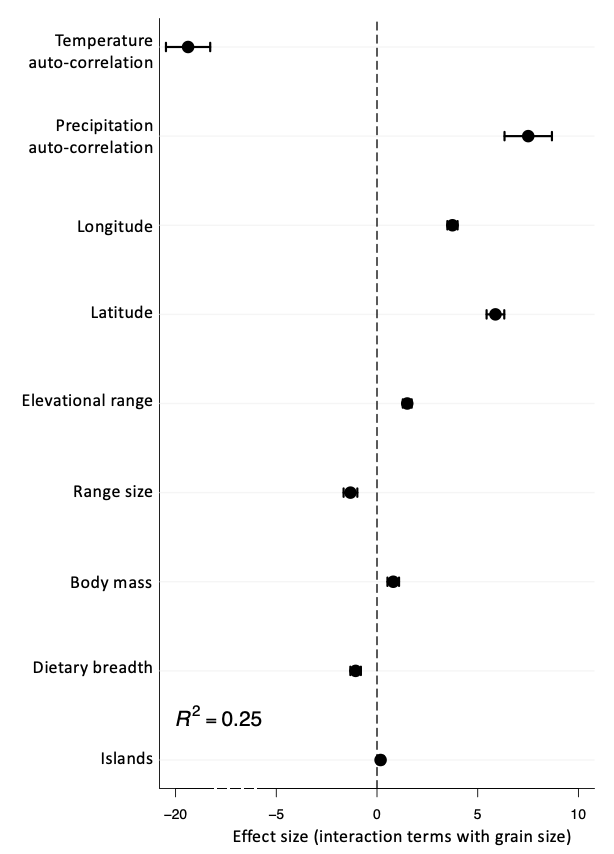


Extended Data Figure 11. The relative effects of nine hypothesized drivers on the spatial scaling of climate change vulnerability using the unbuffered range maps. Temperature auto-correlation, precipitation auto-correlation, latitude, longitude, and range size are calculated from the unbuffered range maps. Positive value means the larger the predictor is, the more likely the vulnerability is going to increase with grain size. Negative value means the larger the predictor is, the less likely the vulnerability is going to decrease with grain size. All nine predictors are combined in a single model.
